# Factors influencing feline antiparasitic prescriptions by veterinarians and demographics across parts of the UK

**DOI:** 10.1002/vro2.70042

**Published:** 2026-09-01

**Authors:** Rosie Elizabeth Hobbs, Aneta Duszak‐Kowal

**Affiliations:** ^1^ School of Veterinary Medicine University of Surrey Guildford UK

## Abstract

**Background:**

Antiparasitic drugs used in companion animals may persist in the environment, contributing to ecological contamination and raising One Health concerns. Understanding behavioural patterns of veterinarians’ prescription is essential for development of effective antiparasitic stewardship programmes.

**Methods:**

A survey of 29 questions was completed between 22 February and 6 March 2024 by 16 qualified veterinary surgeons selected through non‐probability convenience sampling. The online platform JISC enabled creation and distribution of the survey. Descriptive statistics including percentages for the Likert‐scale questions were used to explore nominal and ordinal data alongside the collection of demographic data.

**Results:**

Ease of administration was a primary consideration for 81% of respondents and client preference strongly influenced 44% of veterinarians in product selection. Only 6% of respondents agreed that environmental contamination and drug resistance guided their prescribing. Prophylactic administration was recognised as the most effective strategy by 63% of respondents. Notably only 38% of participants actively kept up to date with parasite treatment guidelines.

**Limitations:**

The sample size was 16 veterinarians and the geographic distribution of participants was limited.

**Conclusions:**

This study highlights regional and demographic factors influencing feline antiparasitic prescribing, emphasising the need for expanded surveillance beyond the studied UK regions. Despite growing concerns about environmental contamination and drug resistance, these factors have a minimal impact on antiparasitic prescribing practices.

## INTRODUCTION

Concerns about the effect of the use of parasiticides on the wider environment has increased over the decade.^1^, ^2^A recent study found that fipronil and imidacloprid persist in UK freshwaters at concentrations harmful to aquatic organisms^3^, ^4^ for up to 60 days.^5^ Understanding prescribing behaviour is essential for developing targeted interventions that maintain antiparasitic treatment efficacy.^6^ The implementation of stewardship programmes is crucial for ensuring responsible prescribing practices as has been shown with antimicrobials.^7^, ^8^ Furthermore, veterinarians' awareness of the broader consequences of antiparasitic use remains largely unexplored.

Antiparasiticides impact all aspects of the One Health triad, including environmental contamination, drug resistance in veterinary patients and zoonotic transmission to humans.^7^, ^9^ Alongside the paucity in drug development,^10^ these factors necessitate judicious prescribing practices. A transdisciplinary approach to antiparasitic prescription is vital for achieving this goal, providing a broader knowledge base, considering animals, humans and the environment and supports universal health control policy.^7^ This approach can enhance understanding of zoonotic disease transmission between animals and humans and reinforces the need for proactive intervention.^7^ Responsible veterinary prescribing is a key strategy for mitigating these risks. However, industry‐level change is necessary to yield significant benefits.^11^

Despite growing evidence of environmental contamination and the negative effect on the insect population caused by antiparasitic drugs, the Veterinary Medicines Directorate does not currently require environmental safety testing for small animal antiparasitics.^12^, ^13^ Research has demonstrated that imidacloprid usage contributes to a deficiency in 69% of bioelements essential to honey bee health.^14^

Antiparasiticide resistance poses a significant public health threat, and drugs used prophylactically in animals often overlap with those used in human medicine.^7^ COVID‐19 increased public awareness of the importance of integrated health policies, shifting perceptions towards One Health principles.^7^ Effective antiparasitic control requires attention to environmental hygiene, individualised risk assessment and proper husbandry practices. Communication and education of clients about these factors is integral to antiparasitic stewardship and can significantly improve compliance.^15^, ^16^ With over 50% of the global population owning pets, incorporating client perspectives into decision making represents an opportunity to provide guidance and education of pet owners on zoonotic risks^16^ alongside the environmental impact, a vital component of antiparasitic stewardship.

Antibiotic stewardship programmes have proven effective in reducing overuse and inappropriate prescriptions of antimicrobials,^8^ suggesting that similar antiparasitic initiatives could prove beneficial. By analysing veterinary prescribing patterns, these programmes provide a foundation for interventions designed to promote behavioural change,^17^ particularly in reducing widespread prophylactic antiparasitic use.^12^ Responsible prescription is essential for maintaining long‐term efficacy and mitigating resistance risk and environmental contamination, with antiparasiticides accounting for 39% of veterinary pharmaceutical products.^18^

Most studies have focused on the use of antiparasiticides usage in livestock, whereas less is known about their use in companion animals. In this context, the present study specifically addresses their use in cats. To the authors’ knowledge, this study is the first to qualitatively investigate the behavioural drivers underlying antiparasitic prescription for cats in the UK. A survey was created and answered by 16 UK‐based veterinarians. The responders were asked to indicate the extent to which they considered different factors when prescribing antiparasitic for cats, including environmental contamination, local parasite distribution, age of animal ease of administration, animal lifestyle, practice of purchasing preferences, client preferences and risk of parasitic resistance.

## METHODS

Participants were recruited via non‐probability convenience sampling through the University of Surrey, School of Veterinary Medicine's partner practice network between 22 February 2024 and 6 March 2024. Royal College of Veterinary Surgeons‐registered veterinary surgeons were invited via an introductory email containing details about the study's objectives and a link to the survey. The inclusion criteria were that participants had prescribed antiparasiticides in the last 4 months preceding the study and that they currently worked in small animal practice. A prize draw was offered as an incentive for participation. Survey data were securely stored within the online survey platform (JISC v3).

The online survey was designed using JISC and structured to investigate factors influencing antiparasitic prescribing behaviour among veterinarians. The survey comprised 29 questions and began with demographic information, including age, gender, county of practice, place of qualification and location of the practice as well as whether they have prescribed antiparasiticides within the last 4 months. Subsequent sections explored prescribing behaviours using a combination of five‐, seven‐ and eight‐point Likert scales and open‐ended free‐text fields to capture qualitative insights.

A pilot study involving four veterinarians was conducted to evaluate the clarity and relevance of the survey items; their responses were excluded from the final dataset. Although the survey included questions related to antiparasitic prescription for both canine and feline patients, data collection and analysis were conducted separately for each species by two independent researchers. The present study focuses solely on prescribing practices for feline patients due to significant differences in lifestyle to canids. Their free roaming nature means that predation is a key route of parasite transmission and significantly increases exposure.^18^, ^19^

Survey responses were exported from JISC to Microsoft Excel (version 16.81) for data familiarisation and storage. Descriptive statistics were applied to analyse the data, which included percentages for the Likert‐scale questions. The dataset comprised nominal variables, including gender and years of experience in practice, as well as ordinal variables (frequency of antiparasitic prescription for routine prevention, frequency with which individual parasite control plans are made, incorporating risk‐based parasite control, frequency of parasite diagnostic testing, and importance placed on regular diagnostic testing), along with demographic information. The survey is included within the Supporting Information and response to open‐ended questions are mentioned within the ‘Results’ section.

## RESULTS

**FIGURE 1:**
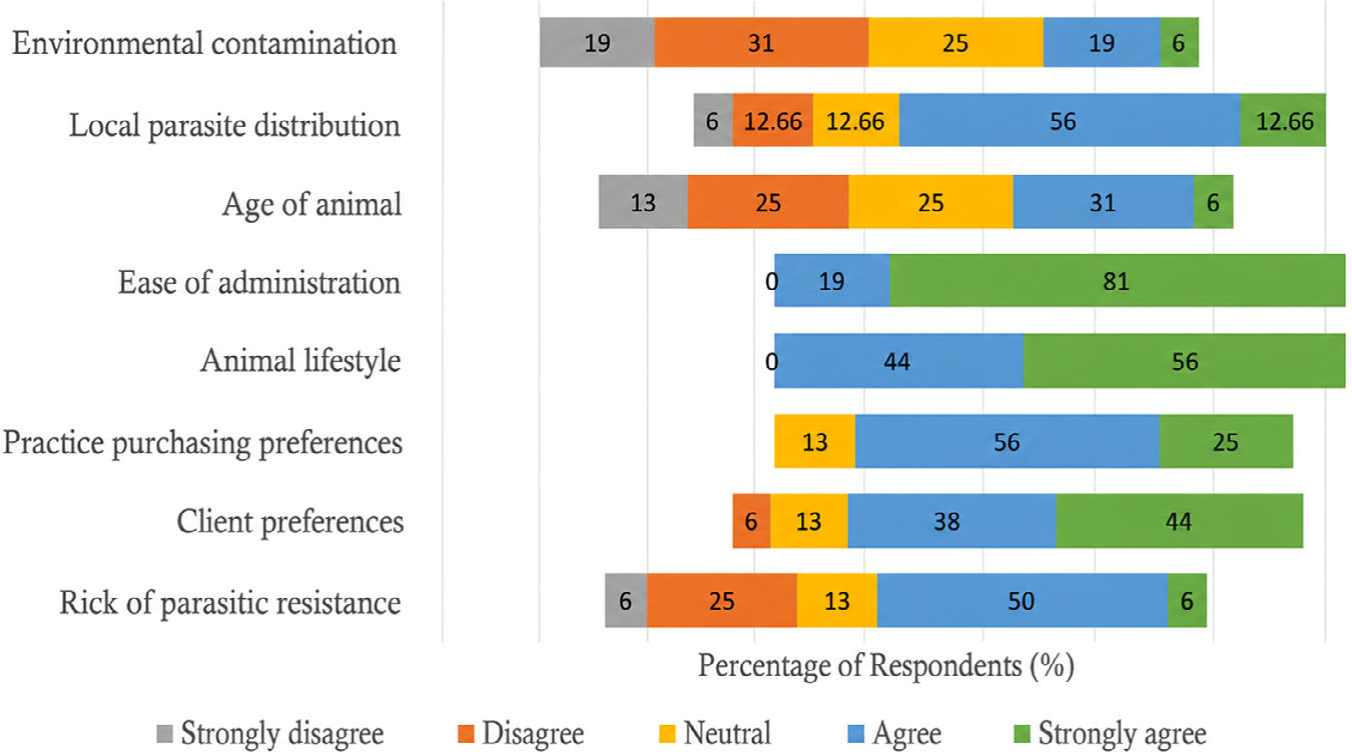
Veterinarians’ perception of factors that influence their prescription of antiparasiticide drugs to cats in clinical practice in the UK, shown as percentage (%) agreement/disagreement (*n* = 16).

A total of 16 responses were received. All participants were registered small animal veterinary practitioners who met the inclusion criteria. Of these, 11 identified as female, four as male and one preferred not to disclose their gender. Ease of administration, animal lifestyle and practice purchasing preferences governed veterinarians’ antiparasiticide prescription. A high proportion of prescribing veterinarians did not consider the risks of environmental contamination and parasitic resistance in the decision‐making process (Figure 1).

A total of 63% of participants considered prophylactic administration of antiparasiticides to be the most appropriate prescription policy for feline patients. Antiparasitic medication was always prescribed by 25% of respondents and was often prescribed by 69%. Two participants reported that the ‘risk of flea infestation was high’ and that infestation could have significant welfare implications.

Although 32% of participants considered regular parasite diagnostic testing to be moderately or very important, only 6% reported routinely performing pre‐treatment diagnostic testing. The frequency of individualised parasite control plans varied; 13% of respondents always implemented such plans, while the majority did so sometimes (38%) or rarely (25%).

Literature addressing the potential risks of parasiticides, particularly within the One Health framework, was read sometimes by 63% of participants and rarely by 25%. Additionally, 50% of respondents disagreed with the statement: ‘There is sufficient knowledge and understanding about the prevention and control of parasite infections in companion animals’.

Among the antiparasitic products reported, Advocate (imidacloprid and moxidectin) and Droncit (praziquantel) were the most commonly prescribed, cited by 88% and 81% of respondents, respectively, while Milpro was not prescribed by any participant (Table 1).

**TABLE 1:** Most commonly prescribed antiparasitic medications to cats by small animal veterinarians practicing in the UK (*n* = 16).

Brand name (active ingredient)	Percentage of respondents (%)
Milpro (praziquantel and milbemycin oxime)	0
Other	6
Stronghold (selamectin and sarolaner)	6
Broadline (eprinomectin and fipronil)	6
Nexguard Combo (praziquantel)	13
Panacur (fenbendazole)	38
Credilo (lotilaner)	63
Droncit (praziquantel)	81
Advocate (imidacloprid and moxidectin)	88

## DISCUSSION

This study aimed to explore veterinarians' antiparasiticide prescribing behaviour in small animal practice and to identify key determinants influencing prescription decisions. To date, limited research has focused specifically on feline parasite prophylaxis, despite their high exposure to parasites.^19^ The development of effective behavioural interventions depends on clear understanding of prescribing behaviour.^6^ This issue is particularly relevant given the increasing awareness of environmental contamination from products containing imidacloprid,^12^ fipronil^20^ and fluralaner, the latter of which is excreted in faeces for several weeks.^21^ While much of the existing research has centred on antibiotic prescription, the principles underlying behavioural intervention are transferable to parasitology. In developed countries, 50% of individuals live with a companion animal,^22^ which highlights the significance of veterinarians’ role in parasite control to safeguard human health (especially those potentially at higher risk of disease) and animal welfare.

Pet owners, practice preferences and ease of administration were identified as significant factors influencing antiparasiticide prescription for cats. A total of 95% of participants reported regularly prescribing topical antiparasitic medications. Ease of administration plays a critical role in feline medicine: topical products such as collars, flea spot‐ons and bedding have been identified as sources of contamination.^12^, ^13^ Further research into the efficacy of alternative control approaches to prophylactic antiparasiticide administration is needed.

Client preferences also influenced veterinarians' product choice. While accommodating owner preferences may enhance compliance, veterinarians should consider the risks of resistance development and environmental contamination. Most research in this area has been conducted within farm and equine medicine, limiting the ability to draw conclusions about the environmental impact of feline antiparasiticides.^23^, ^24^ Due to current insufficient evidence, the environmental risk posed by antiparasitic treatments is poorly understood and further studies are needed to quantify the levels and characterise the routes of environmental exposure, as well as identify any resulting environmental harm.^18^

Without awareness of resistance patterns, it is challenging to implement appropriate mitigation strategies.^22^ In the present study, 69% of respondents indicated that local parasite distribution influenced their prescription choices and veterinarians expressed a desire for further training and education in antimicrobial stewardship programmes. Guidelines developed by the European Scientific Counsel for Companion Animal Parasites may be used to aid prescription based on individual risk and reduce ‘the indiscriminate prophylactic use’ of these products.^12^ However, the available literature on antiparasitic guidelines and One Health principles was not widely read among participants. This lack of engagement is concerning as it may reflect broader gaps in professional awareness of these issues.

This study is limited by the small sample size of participating veterinarians. The response rate could not be calculated, as the number of veterinarians within each partner practice was unknown. Participants had varying levels of clinical experience, and the extent to which this influenced perceptions of resistance, environmental contamination, or the significance of this topic within a One Health framework remains unclear. Additionally, 11 of the 16 respondents practiced in Kent; therefore, the findings may not be representative of the wider UK veterinary profession. Caution should be exercised when extrapolating these results, given the complexity of decision making in clinical practice. Given the importance of this subject, further studies involving wider respondents not only in the UK but also abroad are urgently needed to enhance One Health stewardship.

## CONCLUSION

This study demonstrated that despite environmental contamination and antiparasitic resistance which continue to emerge as critical issues, prioritisation of convenience and client satisfaction was found to be common in veterinary practice. To effectively address these challenges, antiparasitic stewardship strategies must incorporate not only evidence‐based guidelines but also behavioural interventions that target prescribing veterinarians. Veterinarian education may contribute to future strategic parasite control efforts. Further research including larger and international respondent pool is recommended to enhance scientific contribution. The development of an industry‐wide stewardship programme to inform veterinarian decision making may provide benefits in protecting human and animal health and limiting environmental impact.

## AUTHOR CONTRIBUTIONS

Rosie Elizabeth Hobbs performed the literature search, data collection and initial writing of the paper. Aneta Duszak‐Kowal was a significant contributor in editing the paper and provided expertise in the topic area. Both the authors agreed upon the final version of the manuscript submitted to the publisher.

## CONFLICTS OF INTEREST

The authors declare they have no conflicts of interest.

## ETHICS STATEMENT

Ethical approval for this study was obtained from the University of Surrey, School of Veterinary Medicine. No personal or identifiable data were collected, ensuring respondent confidentiality. Informed consent was obtained before survey participation. A prize draw was included to incentivise completion (SageHDR: 1046015‐1045997‐116939968).

## INFORMED CONSENT

Informed consent was obtained from all participants prior to their participation in the survey.

## Supporting information



Supporting Information

## Data Availability

Additional raw data from the survey are available by emailing a.duszak‐kowal@surrey.ac.uk.
