# Supplementary material for: Factors influencing feline antiparasitic prescriptions by veterinarians and demographics across parts of the UK
Source: Vet Rec Open. 2026 Sep 1;13(2):e70042. doi: 10.1002/vro2.70042 (PMC13531695; doi:10.1002/vro2.70042)

We'd love your feedback! Share your thoughts on this new version of the PDF export.

Download

Antiparasitic treatment in companion animals

1. Do you wish to participate in this study? *

Responses: 16

- I wish to participate.: 100%
- No, I don't to participat...

2. Have you prescribed antiparasitics in the last 4 months? *

Responses: 16

- Yes: 100%
- No

3. How many years of clinical experience do you have? *

Responses: 16

- < 2 years: 19%
- 3-5 years: 31%
- 6-10 years: 13%
- 11-15 years: 25%
- 16-20 years
- >21 years: 13%

4. What is your gender? *

Responses: 16

- Female: 69%
- Male: 25%
- Prefer not to say.: 6%

5. Please state which county you practice in?

Responses: 16

Kent

Essex

Kent

kent

Kent

England

UK

Kent

united kingdom

UK

Kent

Kent

Kent

Kent

Kent

Kent

6. Please choose which antiparasitic (endo & ecto) medications you most commonly prescribe in practice for cats (you may select multiple).

Responses: 16

- Panacur (Febendazole): 38%
- Broadline (Eprinomectin, ...: 6%
- Stronghold (Selamectin, S...: 6%
- Droncit (Praziquantel): 81%
- Milpro (Praziquantel, Mil...
- Nexgard Combo (Praziquant...: 13%
- Advocate (Imidacloprid, M...: 88%
- Credilo (Lotilaner): 63%
- Other: 6%

7. Please choose which antiparasitic medications (endo/ecto) you most commonly prescribe in practice for dogs (you may select multiple).

Responses: 16

- Advocate (Imidacloprid, M...: 88%
- Droncit (Praziquantel): 81%
- Drontal (Febantel, Pyrant...: 75%
- Panacur (Fenbendazole): 63%
- NexGard Spectra (Afoxolan...: 50%
- Other: 44%
- Bravecto (Fluralaner): 25%
- Milbemax (Milbemycin Oxim...: 13%
- Stronghold (Selamectin): 6%
- Seresto collars (Flumethr...: 6%
- Nextguard (Afoxolaner): 6%
- Simparica Trio (Sarolaner...
- Milpro (Milbemycin Oxime...

8. How far do you agree with this statement: When considering treating a cat for ectoparasites and endoparasites, I consider... *

Responses: 16

8.1 Risk of parasitic resistance

- Strongly Disagree: 6%
- Disagree: 25%
- Neither agree nor disagre...: 13%
- Agree: 50%
- Strongly Agree: 6%

8.2 Client Preferences

- Strongly Disagree
- Disagree: 6%
- Neither agree nor disagre...: 13%
- Agree: 38%
- Strongly Agree: 44%

8.3 Practice Purchasing Preferences

- Strongly Disagree
- Disagree
- Neither agree nor disagre...: 13%
- Agree: 56%
- Strongly Agree: 25%

8.4 Animal Lifestyle (outdoor/indoor cat)

- Strongly Disagree
- Disagree
- Neither agree nor disagre...
- Agree: 44%
- Strongly Agree: 56%

8.5 Ease of administration (drug formulation).

- Strongly Disagree
- Disagree
- Neither agree nor disagre...
- Agree: 19%
- Strongly Agree: 81%

8.6 Age of animal

- Strongly Disagree: 13%
- Disagree: 25%
- Neither agree nor disagre...: 25%
- Agree: 31%
- Strongly Agree: 6%

8.7 Local parasite distribution

- Strongly Disagree: 6%
- Disagree: 13%
- Neither agree nor disagre...: 13%
- Agree: 56%
- Strongly Agree: 13%

8.8 Environmental Contamination (aquatic water systems)

- Strongly Disagree: 19%
- Disagree: 31%
- Neither agree nor disagre...: 25%
- Agree: 19%
- Strongly Agree: 6%

9. Beyond those mentioned, are there any other factors which influence your choice of product when prescribing to feline patients?

Responses: 4

Numbers of animals in a household and species of animals cohabitating. Amy underlying skin disease such as allergies that make ruling out parasite infestations more important.

Frequency of flea infestations, or previous treatments that the cat has been on in the past. How effective any previous flea products have been in managing fleas

Young children who may be contaminated by stroking the pet after application of topical product

previous adverse reactions ie skin irritation

10. How far do you agree with this statement: When considering treating a dog for ectoparasites and endoparasites, I consider... *

Responses: 16

10.1 Risk of parasiticide resistance

- Strongly disagree: 6%
- Disagree: 19%
- Neither agree nor disagre...: 13%
- Agree: 50%
- Strongly agree: 13%

10.2 Client preference

- Strongly disagree: 6%
- Disagree
- Neither agree nor disagre...: 13%
- Agree: 44%
- Strongly agree: 38%

10.3 Practice purchasing preferences

- Strongly disagree
- Disagree: 6%
- Neither agree nor disagre...: 6%
- Agree: 63%
- Strongly agree: 25%

10.4 Risk of environmental contamination (e.g. risk to wildlife, water contamination, ect.)

- Strongly disagree: 13%
- Disagree: 25%
- Neither agree nor disagre...: 19%
- Agree: 38%
- Strongly agree: 6%

10.5 Lifestyle of the dog

- Strongly disagree
- Disagree
- Neither agree nor disagre...
- Agree: 63%
- Strongly agree: 38%

10.6 Parasite distribution in your local area

- Strongly disagree
- Disagree
- Neither agree nor disagre...: 6%
- Agree: 50%
- Strongly agree: 44%

11. Other than those mentioned above, are there any other factors that influence the choice and frequency of parasiticides you prescribe to dogs?

Responses: 5

Any cats living in the home, I find dogs living in a household with cats are more likely to get fleas, if there are not any cats I wouldn't routinely give flea prevention unless the dog has skin allergies. The high incidence of lungworm in our area makes prophylactic anthlemintic use important.

Previous treatments used and how efficacious managing fleas have been for the client

Same as for cats

Ease of administration - a lot prefer oral meds now rather than spot on

High prevalence of lungworm in our area

12. Please indicate the most common way you administer antiparasitic medication to cats? *

Responses: 16

- Oral: 6%
- Topical (including collar...: 94%

13. Please indicate the most common way you administer anti-parasitic medication to dogs? *

Responses: 16

- Oral: 44%
- Topical (including spot- ...: 56%

14. Please indicate how often you carry out pre-treatment parasite diagnostic testing in feline patients? *

Responses: 16

- Never: 44%
- Rarely: 31%
- Sometimes: 19%
- Often: 6%
- Always

15. Please indicate how often you carry out pre-treatment parasite diagnostic testing in canine patients? *

Responses: 16

- Never: 38%
- Rarely: 31%
- Sometimes: 25%
- Often: 6%
- Always

16. If there is a difference in your answer for feline and canine diagnostic testing please state why.

Responses: 2

N/A

Flea comb and lungworm testing are the only/main diagnostic tests used

17. Please indicate the level of importance you see in regular parasite diagnostic testing? *

Responses: 16

- not at all important
- low importance: 13%
- slightly important: 31%
- neutral: 25%
- moderately important: 13%
- very important: 19%
- extremely important

18. Please indicate how often you prescribe anti-parasitic medication for routine prevention in dogs?

Responses: 16

- Never
- Rarely
- Sometimes: 6%
- Often: 44%
- Always: 50%

19. Please indicate how often you prescribe anti-parasitic medication for routine prevention in cats?

Responses: 16

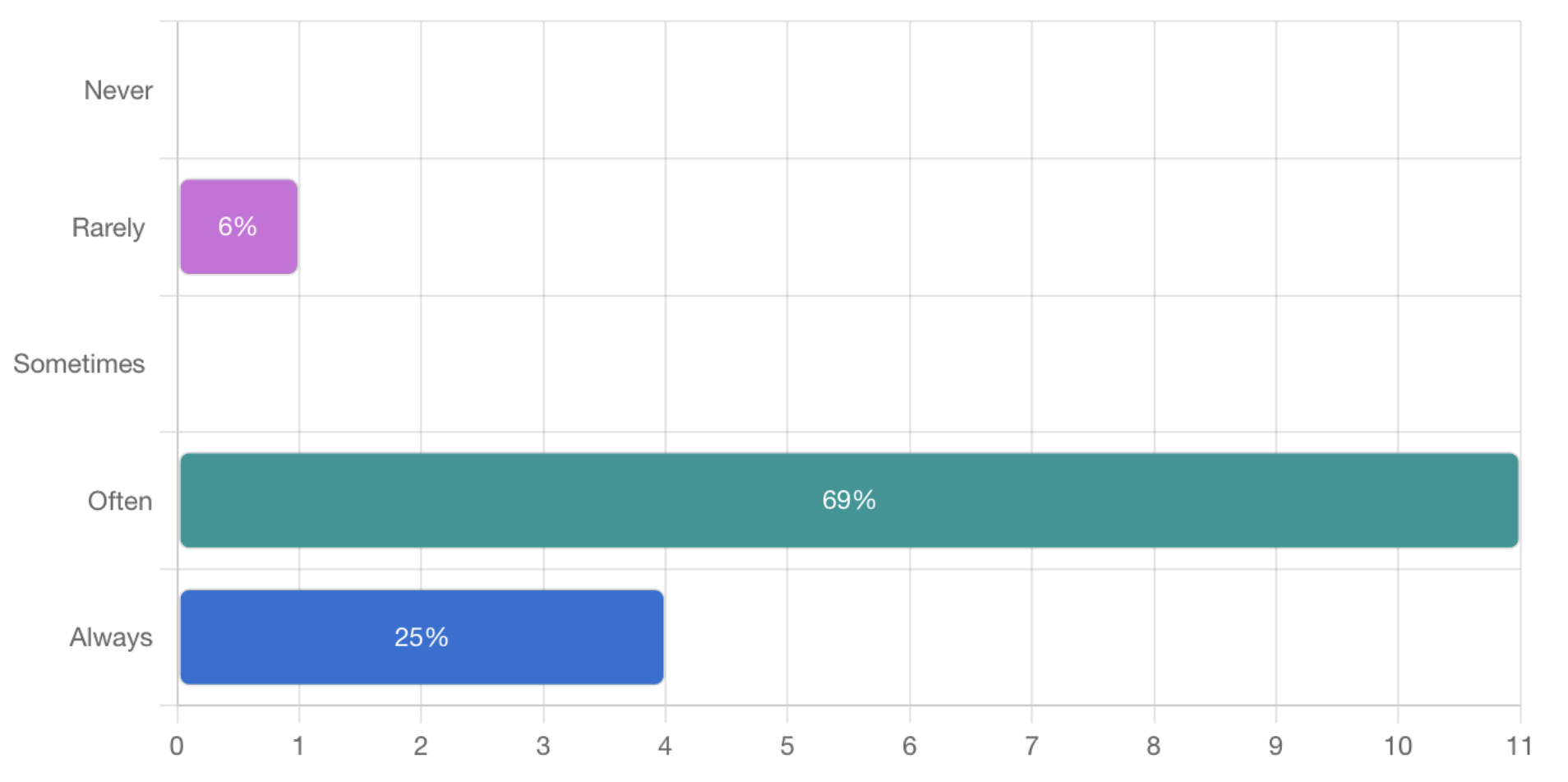

20. Please indicate which of the below options do you believe is more appropriate when prescribing anti-parasitic drugs to feline patients?

Responses: 16

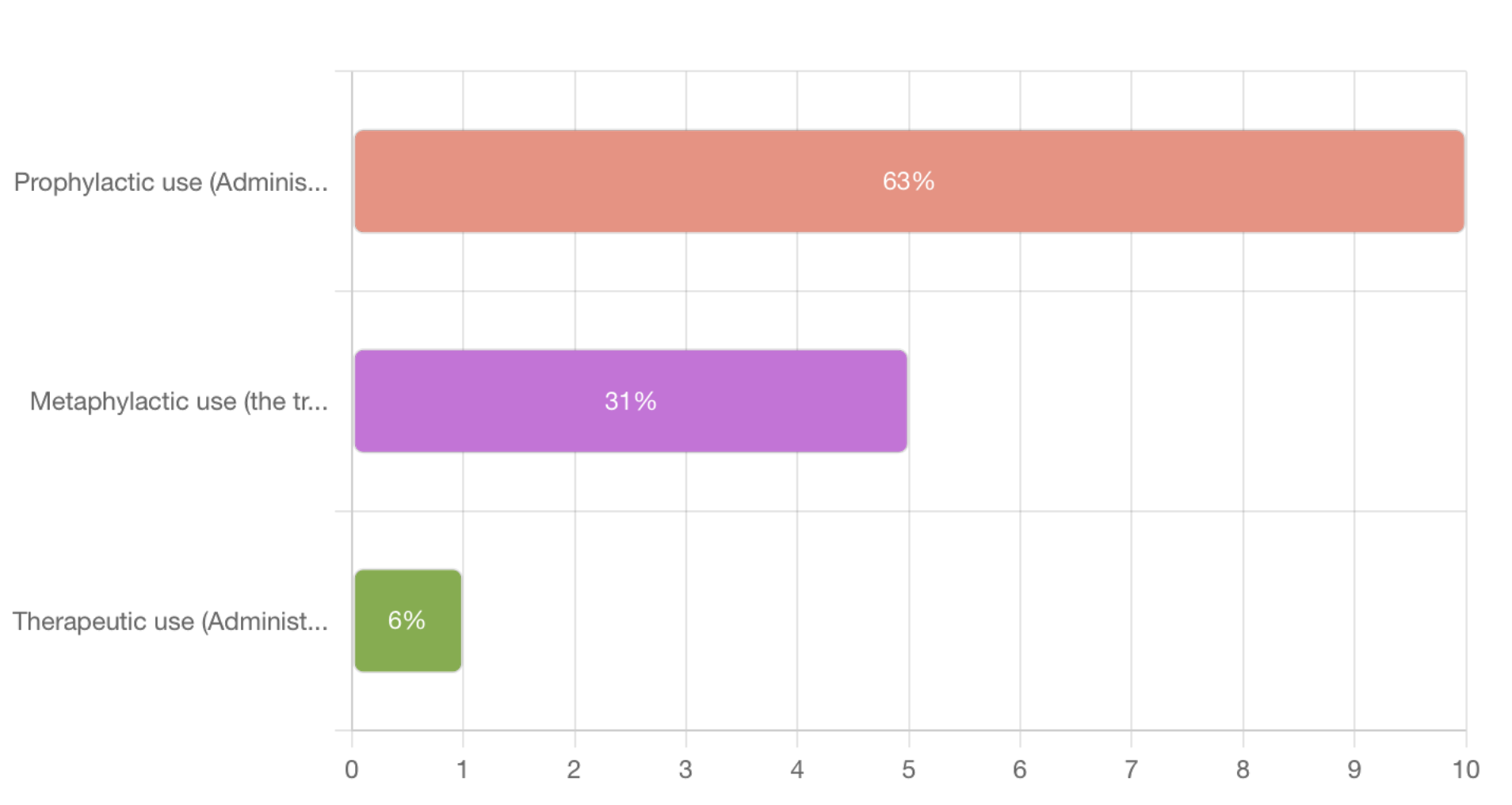

21. If you chose prophylactic use, please indicate why:

Responses: 8

With outdoor cats, risk of flea infestation is high.

issues with fleas once an infestation takes hold outweigh prophylactic use

Owners can be bad at identifying parasites in their pets leading to poorer welfare

Outdoor cats are at high risk for catching fleas - from other cats, properties, wildlife etc. Their roaming range is broad enough that it is presumed they have a high exposure to parasites. They are also in contact with other animals that may or may not be on parasite treatment. As such I've seen more outdoor cats who are on prescription flea treatment still presenting with fleas in their coat or flea allergy dermatitis.

Prevention of disease.

Risk of ill health from parasite infestation

If outdoor cats, flea treatment can be much more difficult than preventation. Can cause significant disease is flea allergy present.

Owners hate fleas

22. Please indicate which of the below options do you believe is more appropriate when prescribing anti-parasitic drugs to canine patients?

Responses: 16

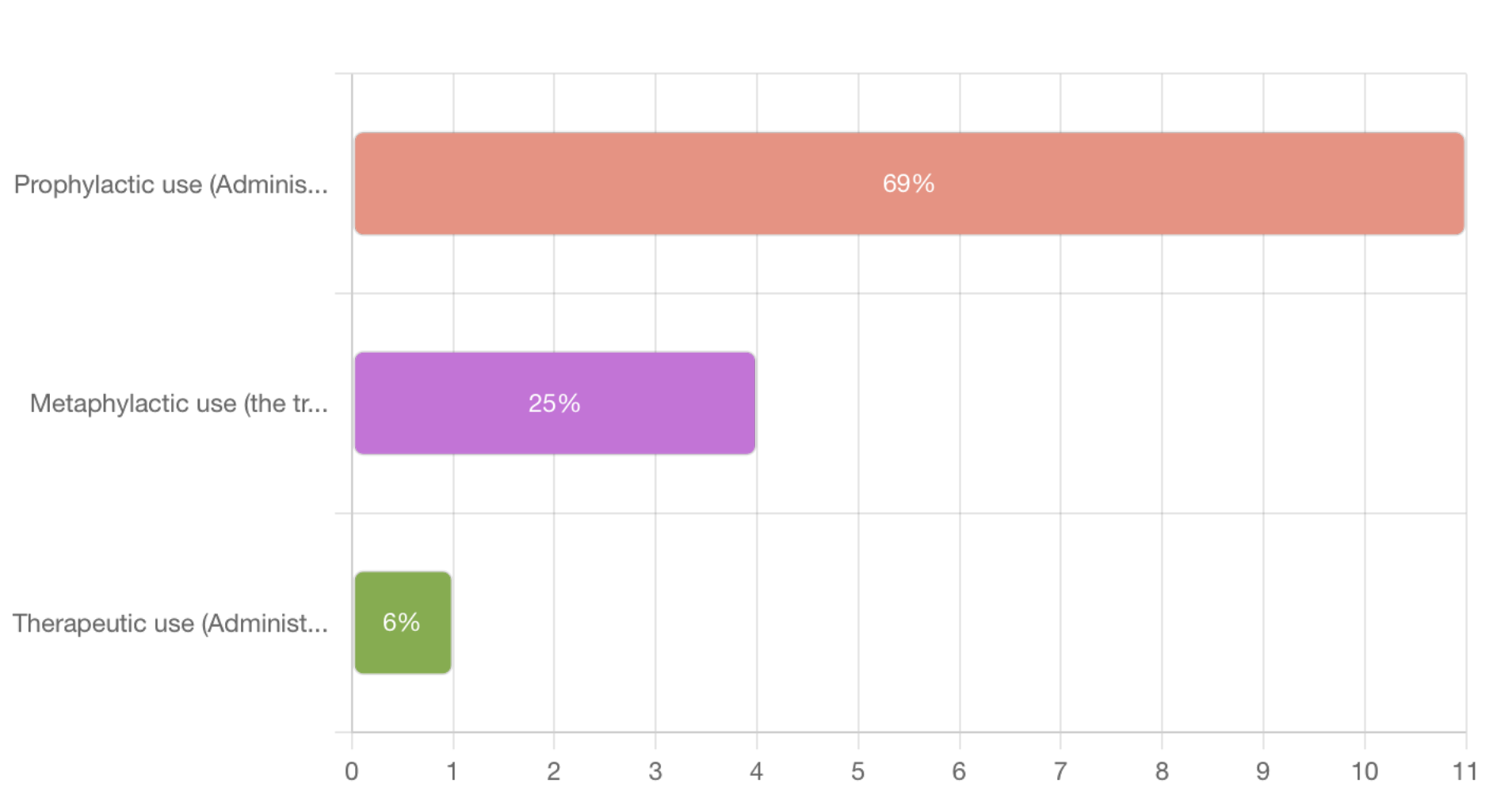

23. If you chose prophylactic use, please indicate why:

Responses: 10

My main focus in canine patients is prevention of Angiostrongylus vasorum infestation,

We have a very high local lung worm problem and see clinical cases regularly.

fatal nature of things like lungworm where the risk of not treating prophylactically outweighs other considerations

Risk of resistance is low due to the residue population of parasites in wild animals. The consequences of parasites such as lungworm can be profound.

Lungworm can kill and is prevalent

Prevention of disease

risk of ill health from parasite infestation - particularly lungworm

Lungworm is common in the local area and can be fatal

HIgh risk of lungworm and potentially life threatening nature of lungworm if they were to get it,

Unfortunately we do not have readily available and cheap diagnostic tests for lungworm in dogs. Ideally we would have a test that could be run on owner submitted samples rather than having to blood test them.

24. Do you keep up to date with existing veterinary parasitic guidelines (E.g European Scientific Counsel Companion Animal Parasites documents). *

Responses: 16

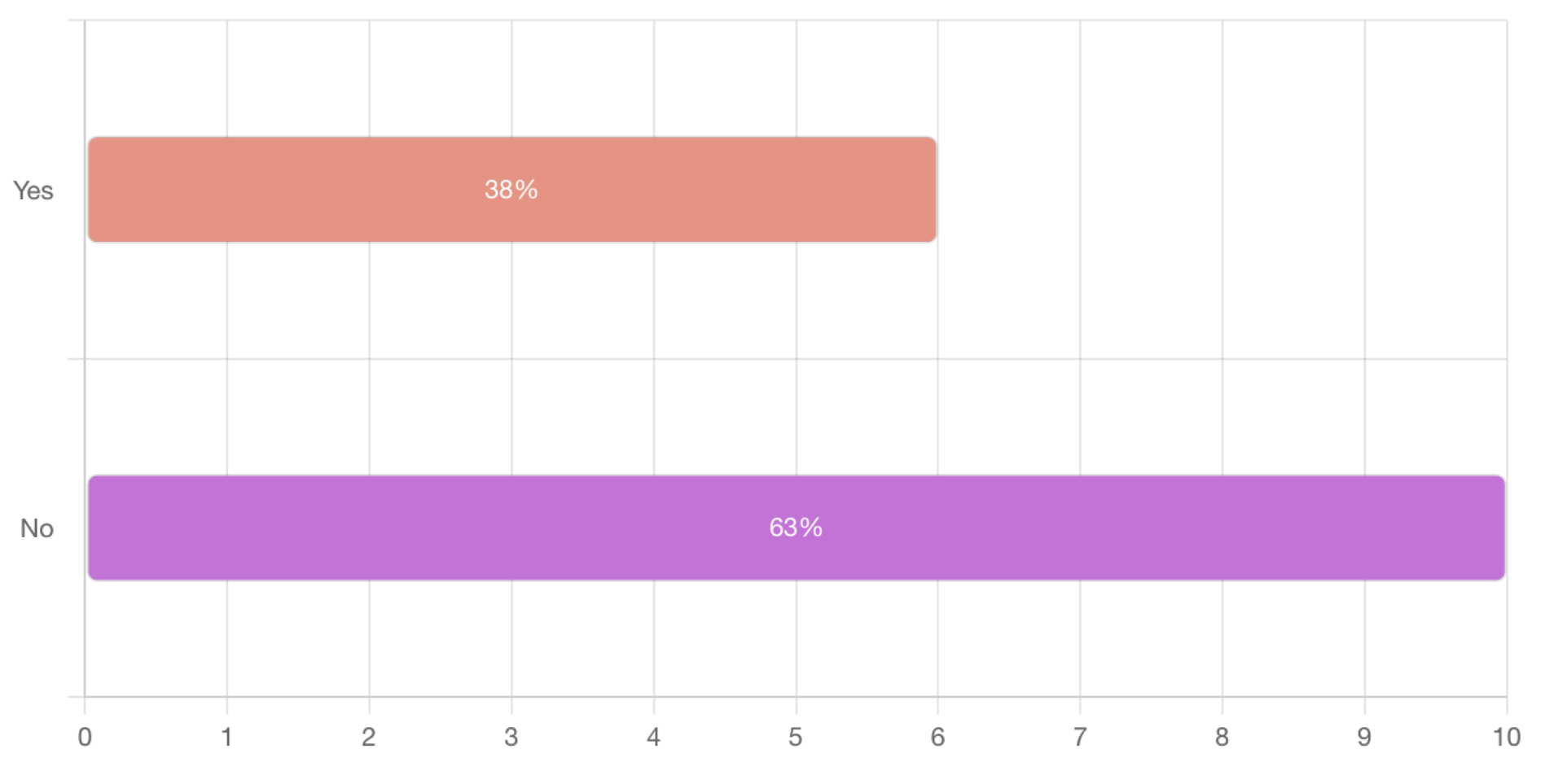

25. How often do you keep up to date with published papers/literature around the potential risks of parasiticides affecting One Health? *

Responses: 16

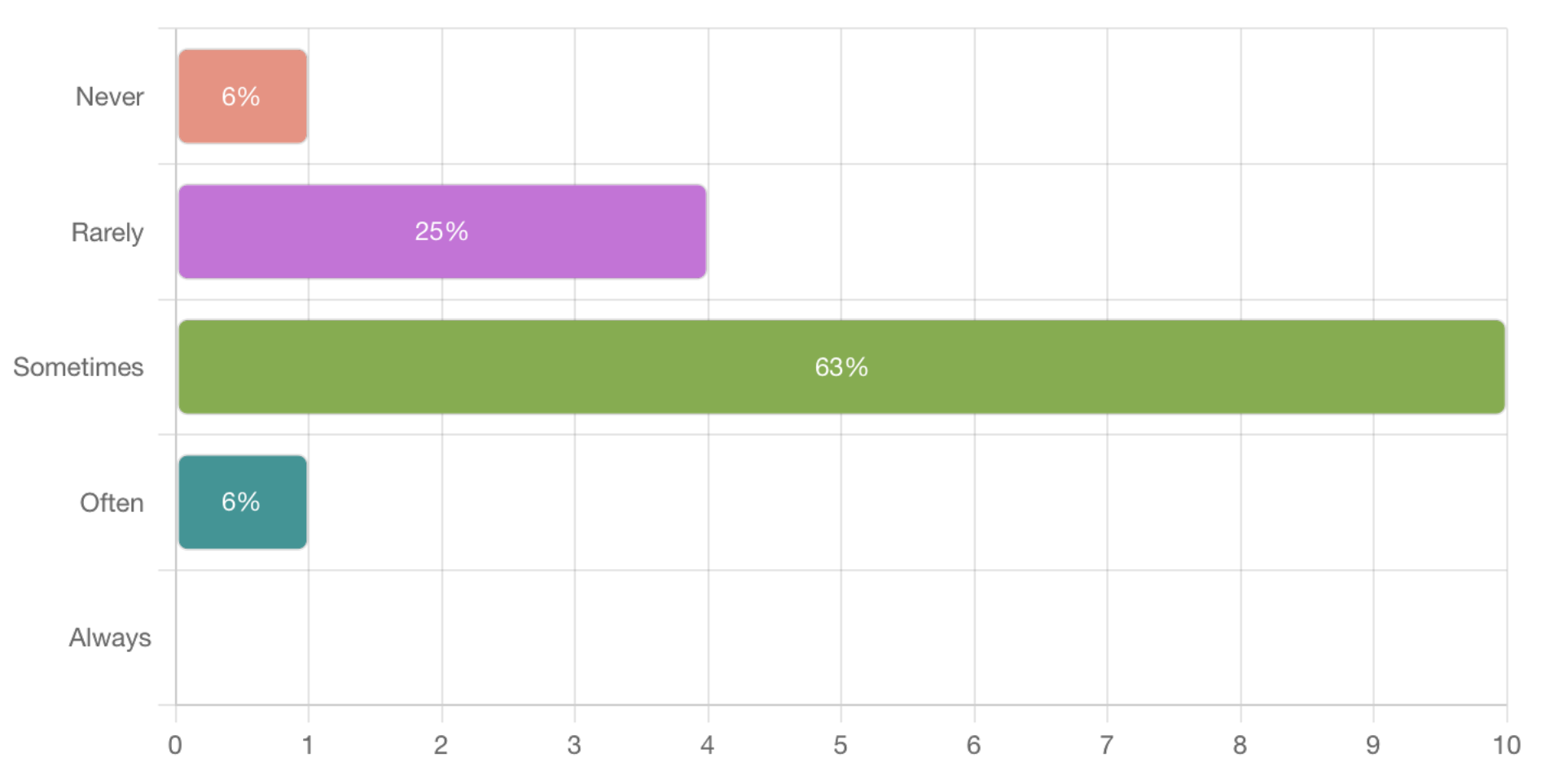

26. How often do you make individual parasite control plans for your canine patients incorporating a risk-based parasite control? (e.g. taking into account lifestyle factors of the dog, ect.)

Responses: 16

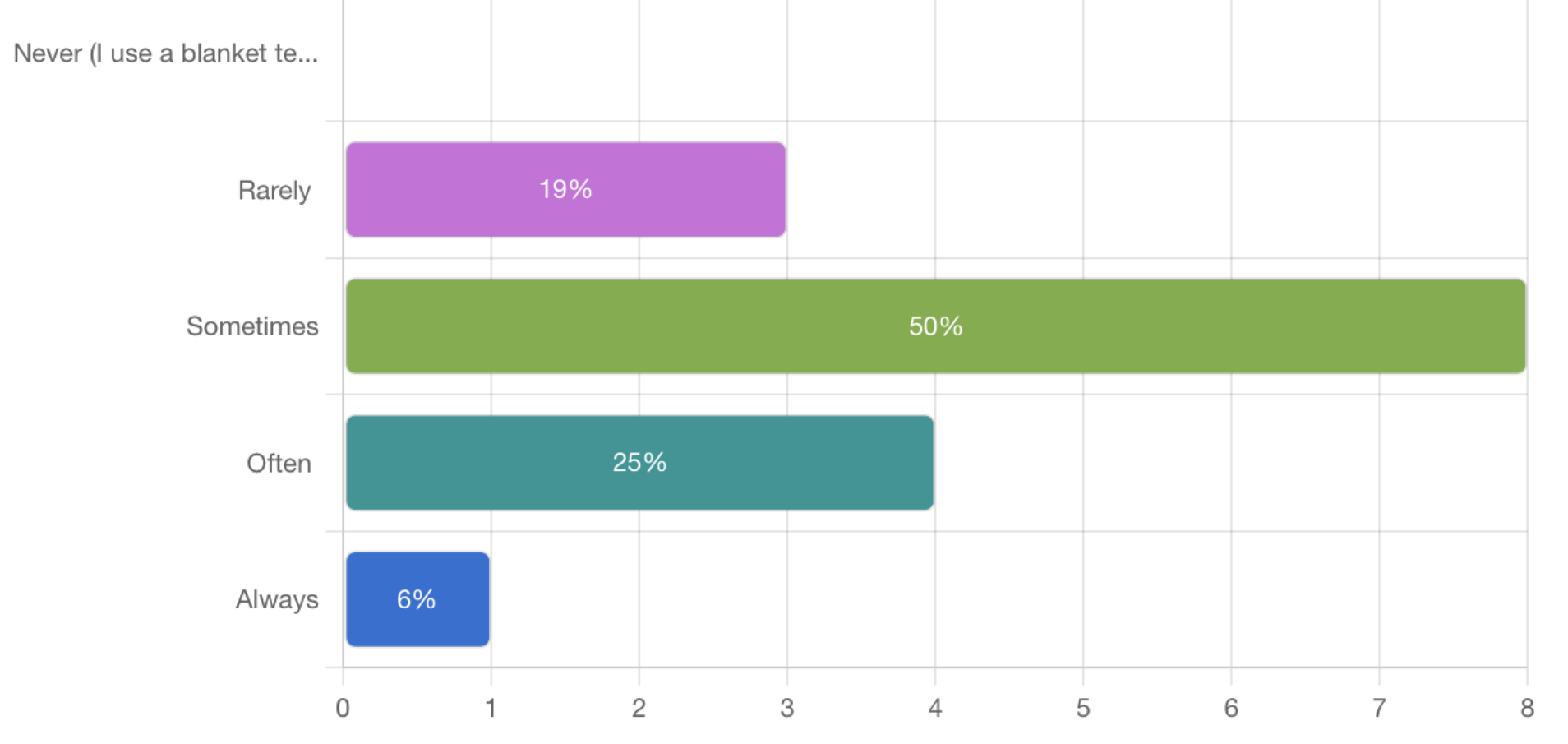

27. How often do you make individual parasite control plans for your feline patients, incorporating risk-based parasite control (based on local parasite fauna & cat's lifestyle)?

Responses: 16

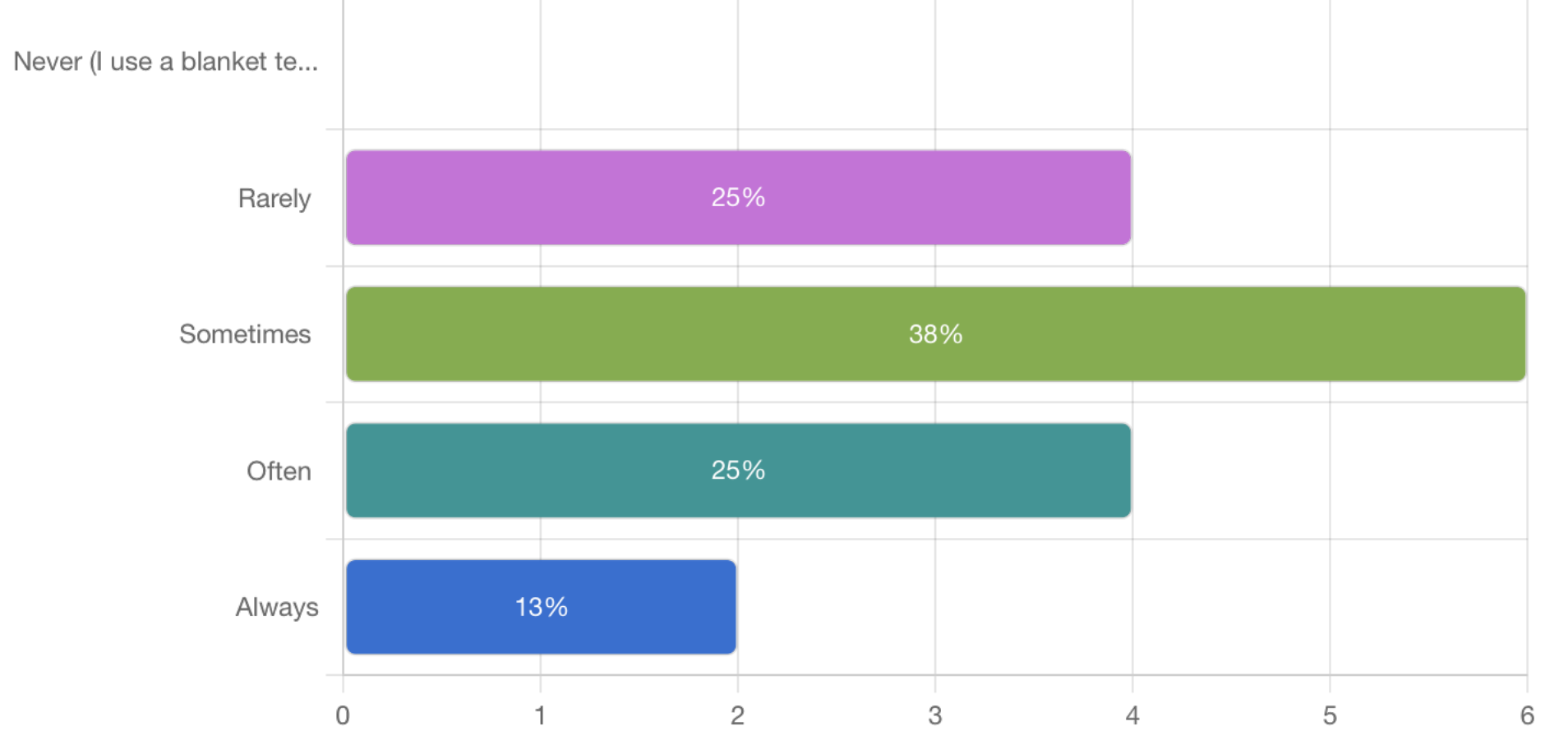

28. Do you believe you have autonomy in choosing antiparasitic medications?

Responses: 16

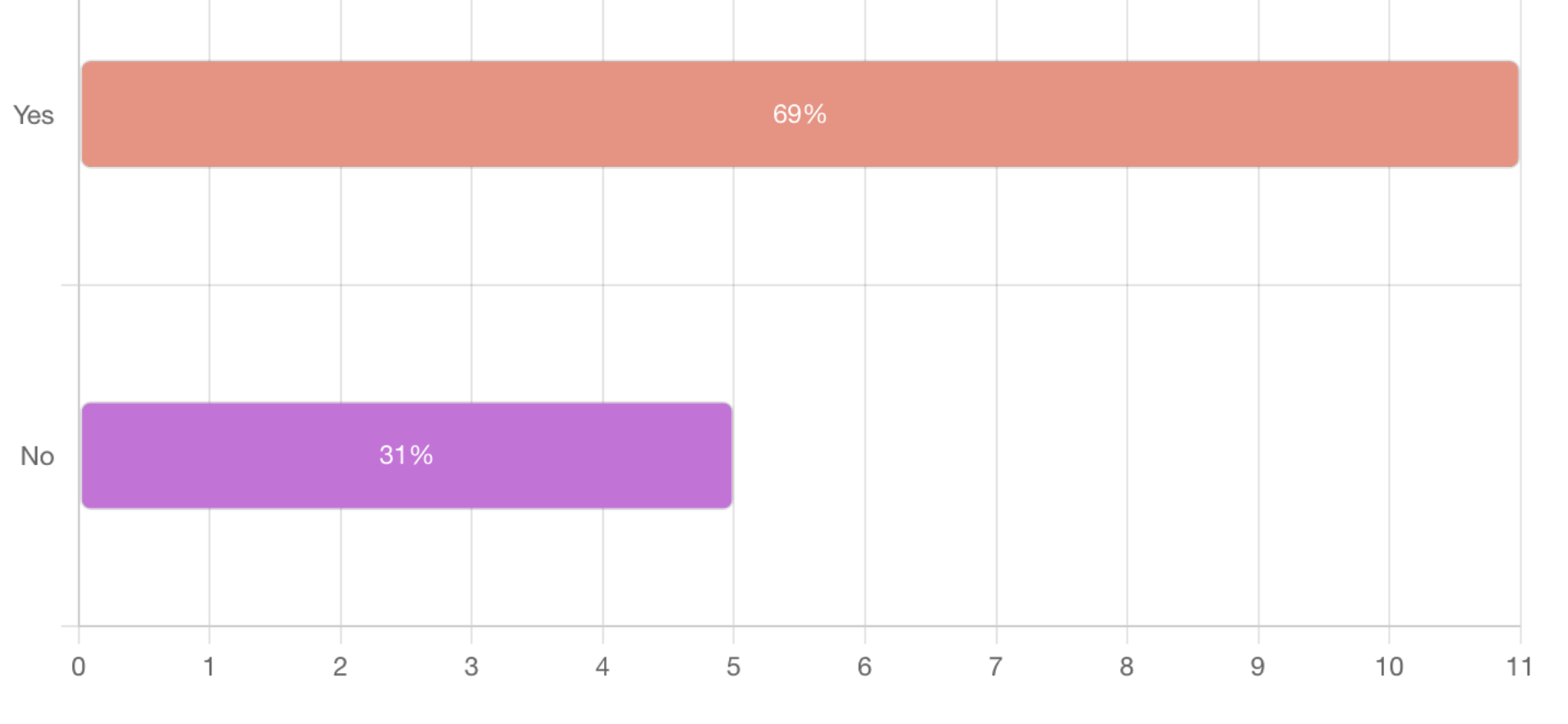

29. How far do you agree with this statement: "There is sufficient knowledge and understanding about prevention and control of parasite infections in companion animals?" *

Responses: 16

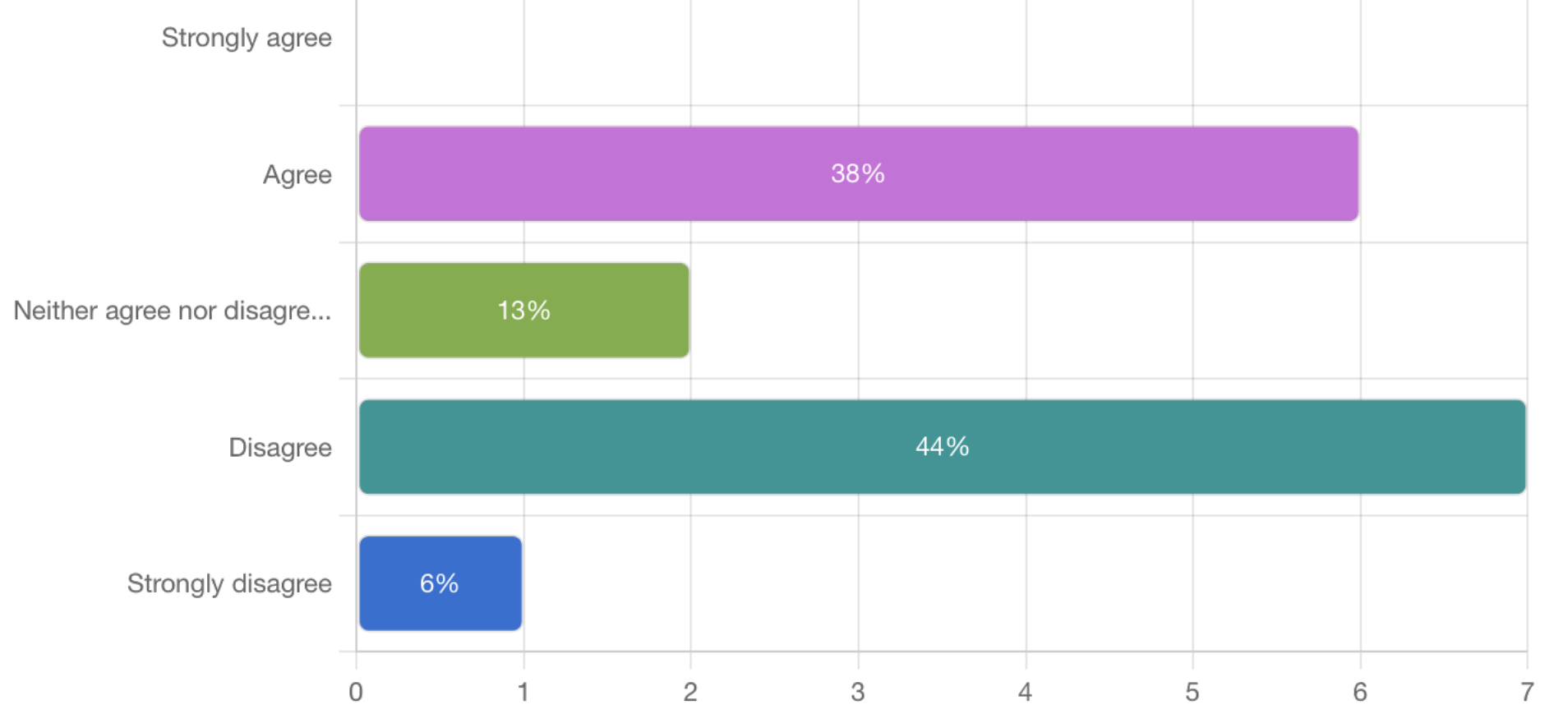

Supplement: Supplementary file 1 [file VRO2-13-e70042-s001.pdf]
